# Practical Intestinal Surgery

**Published:** 1891-12

**Authors:** 


PRACTICAL INTESTINAL SURGERY—By Fred B. Robinson, B. S., M. D. Professor Anatomy and Clinical Surgery Toledo Medical College. Geo S. Davis, Detroit, Mich., 1891.

This is the second Volume on the subject by the same author.

Chapter XIV. is devoted to the subject of Gastro-Enterostomy, in which the author gives the very interesting details of the operation for making an artificial opening from the stomach into the intestines, whereby the difficulties attending disease attending the pyloric orifice are obviated. Here we are brought to realize the vast progress in modern surgical procedures.

The author in discussing the merits of this operation, speaks of it as the favored rival of pylorectomy and gives as the most successful operator Prof. Luecke of Strasburg, who, in *eight* operations was successful in *seven*.

A very interesting point in the author's statements is the development of a sphincter like function in the artificial opening between the stomach and bowel, sufficiently retentive to well take the place of the natural pylorus.

Chapter XVIII is devoted to the discussion of the operation of Gastrostomy, and describes the details of experiments in the operation with their results.

Quite an interesting chapter is given to Jobert's operation in circular enterorrhaphy and the author's modification of the same.

The chapter on Gun Shot Wounds of the intestines is very interesting and instructive from an experimental and statistical point, and altogether the work is an invaluable contribution to the literature of the department to which it belongs.

